# Complex Syndrome of the Complete Occlusion of the End of the Superior Mesenteric Vein, Opposed with the Stable Gastric Pentadecapeptide BPC 157 in Rats

**DOI:** 10.3390/biomedicines9081029

**Published:** 2021-08-17

**Authors:** Mario Knezevic, Slaven Gojkovic, Ivan Krezic, Helena Zizek, Hrvoje Vranes, Dominik Malekinusic, Borna Vrdoljak, Tamara Knezevic, Katarina Horvat Pavlov, Domagoj Drmic, Miro Staroveski, Antonija Djuzel, Zoran Rajkovic, Toni Kolak, Eva Lovric, Marija Milavic, Suncana Sikiric, Ivan Barisic, Marijan Tepes, Ante Tvrdeic, Leonardo Patrlj, Sanja Strbe, Marija Sola, Andrej Situm, Antonio Kokot, Alenka Boban Blagaic, Anita Skrtic, Sven Seiwerth, Predrag Sikiric

**Affiliations:** 1 Department of Pharmacology, School of Medicine, University of Zagreb, 10000 Zagreb, Croatia; mariknezevic@gmail.com (M.K.); slaven.gojkovic.007@gmail.com (S.G.); ivankrezic94@gmail.com (I.K.); zizekhelena@gmail.com (H.Z.); hrvoje.vranes@gmail.com (H.V.); dominikmalekinusic@gmail.com (D.M.); borna.vrdoljak@gmail.com (B.V.); 0101tamara@gmail.com (T.K.); iddrmic@mef.hr (D.D.); miro.staroveski@gmail.com (M.S.); antonija.djuzel@gmail.com (A.D.); tkolak@kbd.hr (T.K.); inbarisic@gmail.com (I.B.); mtepes@gmail.com (M.T.); ante.tvrdeic@mef.hr (A.T.); patrljleo@gmail.com (L.P.); strbes@gmail.com (S.S.); marijasola11@gmail.com (M.S.); andrej_situm@yahoo.com (A.S.); antonio.kokot@mefos.hr (A.K.); abblagaic@mef.hr (A.B.B.); 2 Department of Pathology, School of Medicine, University of Zagreb, 10000 Zagreb, Croatia; katarina.horvat@gmail.com (K.H.P.); eva.lovric@kb-merkur.hr (E.L.); marija.milavic@mef.hr (M.M.); suncanasikiric@gmail.com (S.S.); sven.seiwerth@mef.hr (S.S.); 3 Department of Surgery, Faculty of Dental Medicine and Health, University of Osijek, 31000 Osijek, Croatia; zrajkovi@net.hr

**Keywords:** BPC 157, superior mesenteric vein occlusion, vascular recruitment, rats

## Abstract

**Background**. Gastric pentadecapeptide BPC 157 therapy in rats compensated irremovable occlusion of various vessels and counteracted the consequent multiorgan dysfunction syndromes by activation of the corresponding collateral bypassing loops. Thus, we used BPC 157 therapy against the irremovable occlusion of the end of the superior mesenteric vein. **Methods**. Assessments, for 30 min (gross recording, venography, ECG, pressure, microscopy, biochemistry, and oxidative stress) include the portal and caval hypertension, aortal hypotension, and centrally, the superior sagittal sinus hypertension, systemic arterial and venous thrombosis, ECG disturbances, MDA-tissue increase, and heart, lung, liver, kidney and gastrointestinal tract, in particular, and brain (cortex (cerebral, cerebellar), hypothalamus/thalamus, hippocampus) lesions. Rats received BPC 157 medication (10 µg/kg, 10 ng/kg) intraperitoneally at 1 or 15 min ligation time. **Results**. BPC 157 rapidly activated the superior mesenteric vein–inferior anterior pancreati-coduodenal vein–superior anterior pancreaticoduodenal vein–pyloric vein–portal vein pathway, reestablished superior mesenteric vein and portal vein connection and reestablished blood flow. Simultaneously, toward inferior caval vein, an additional pathway appears via the inferior mesenteric vein united with the middle colic vein, throughout its left colic branch to ascertain alternative bypassing blood flow. Consequently, BPC 157 acts peripherally and centrally, and counteracted the intracranial (superior sagittal sinus), portal and caval hypertension, aortal hypotension, ECG disturbances attenuated, abolished progressing venous and arterial thrombosis. Additionally, BPC 157 counteracted multiorgan dysfunction syndrome, heart, lung, liver, kidney and gastrointestinal tract, and brain lesions, and oxidative stress in tissues. **Conclusion**. BPC 157 therapy may be specific management also for the superior mesenteric vein injuries.

## 1. Introduction

Mesenteric venous thrombosis is an uncommon cause of mesenteric ischemia accounting for 5–15% of the cases [1], now associated also with COVID-19 [2]. With the irremovable occlusion of the rat superior mesenteric vein and stable gastric pentadecapeptide BPC 157 therapy (for review see, e.g., [3,4,5]), we attempt to reveal the specific management of the superior mesenteric vein injuries, the activation of the collateral bypassing loops [6,7,8,9,10], and to resolve the consequent perilous syndrome, and in particular ligation [11]. Therapy of the superior mesenteric vein injuries and consequent syndrome are both presently resolved only partially, due to complications [1,11], and/or at least not fully described in the superior mesenteric vein research.

As therapy for the irremovable occlusion of the rat superior mesenteric vein, BPC 157 goes due to its particular epithelium–endothelium cytoprotective effect on blood vessels functioning [12]. It was theorized that epithelium–endothelium cytoprotective effect results with a huge range of beneficial effects for cytoprotective agent’s activity [12], and for BPC 157 in particular, in the situation of the vessel occlusion [6,7,8,9,10,13,14,15] or injury [16], to the activation of the collateral bypassing loops. BPC 157 activated “bypassing key” [6,7,8,9,10], collateral pathways reliant on the injurious occlusion, inferior caval vein syndrome [6], Pringle maneuver (portal triad temporary occlusion), ischemia, reperfusion [7], Budd–Chiari syndrome (suprahepatic inferior caval vein occlusion) [8], superior mesenteric artery occlusion syndrome [9], and superior sagittal sinus occlusion syndrome [10]. Indicatively, in recovering rats with major central or peripheral vessel occlusion, and consequent multiorgan dysfunction syndrome, BPC 157 is acting both peripherally and centrally [6,7,8,9,10]. Peripherally, the left superior caval vein–azygos vein–inferior caval vein shunt would skip and compensate the suprahepatic occlusion of the inferior caval vein and reestablish blood flow and resolve Budd–Chiari syndrome [8]. The left ovarian vein would resolve the infrarenal occlusion inferior caval vein syndrome [6], the porto-caval shunt compensated the Pringle maneuver obstruction [7], and the inferior anterior pancreaticoduodenal artery and inferior mesenteric artery compensated the superior mesenteric artery occlusion syndrome [9]. In addition, centrally, as therapy, in the rats with the occluded superior sagittal sinus, BPC 157 rapidly recruited (para)sagittal venous collateral circulation [10].

Possibly, this may be relevant in relation with the report of the thrombosis, and thereby, blood stasis, in the patients treated with occluded superior mesenteric vein [1,2,11]. Illustratively, with BPC 157 therapy, attenuated/eliminated Pringle maneuver (portal triad temporary occlusion), ischemia, reperfusion [7], as well as the whole Budd–Chiari syndrome [8] included the counteraction of the prominent deadly syndrome [7,8]. Heart dysfunction, lung lesions (i.e., time-dependent and time-independent features resembling acute respiratory distress syndrome (ARDS) exudative phase features), liver failure, and gastrointestinal lesions, widespread arterial and venous thrombosis, severe portal and caval hypertension and aortal hypotension were all counteracted [7,8]. A similar syndrome, including intracranial hypertension, was noted with the central vein occlusion, the occlusion of the superior sagittal sinus [10], and interestingly, also from the periphery, with the occlusion of the superior mesenteric artery [9]. BPC 157 therapy effect (in addition to the counteracted intracranial (superior sagittal sinus) hypertension, brain swelling and lesions [9,10]) was as in the previous peripheral vessel occlusion studies [6,7,8]. Furthermore, these beneficial effects were ascribed to competition with Virchow’s triad, and its resolution, which could be present [6,7,8,9,10,13,14,15]. In addition, BPC 157 maintains thrombocytes function [17] without interfering with coagulation pathways [17,18,19]. This particular activity was ascribed to BPC 157 interaction with several molecular pathways [6,20,21,22,23,24,25,26,27,28,29], and modulatory effects on prostaglandins- and NO-system [30,31] and on vasomotor tone and the activation of Src–Caveolin-1–eNOS pathway [23], and its action as stabilizer of cellular junctions [22], and free radical scavenger [32,33,34], in particular in vascular occlusion studies [6,7,9,13,14,15,16].

Especially, no study investigated the specific point of the rapid activation of the particular collateral circulation to bypass superior mesenteric vein occlusion and reestablish blood flow to counteract superior mesenteric vein occlusion-induced disturbances. We investigated the broken communication between the superior mesenteric and portal vein, the most proximal point of the superior mesenteric vein as the breaking occlusion point, at the end of the superior mesenteric vein, just below the joining of the lienal vein. As resolution, we investigated the activation of the bypassing loop of the inferior anterior pancreaticoduodenal vein and superior pancreaticoduodenal vein to pyloric vein to reestablish both the communication between the superior mesenteric vein and portal vein and the recovery of their blood flow. Besides, in the rats, caudally, the superior mesenteric vein network is joined to superior and inferior mesenteric veins. This fairly correlates with the patients, which have the inferior mesenteric vein joined with the superior mesenteric vein [35,36]. Thus, as an additional more remote, specific bypassing pathway that should be simultaneously activated, united with the middle colic vein, the inferior mesenteric vein throughout its left colic branch, may fairly contribute to ascertain alternative bypassing blood flow achieved via inferior caval vein. Regularly, providing the body cavity abdominal–thoracic–brain interactions, rapid transmission up through the venous system [37,38], a perilous syndrome similar those described in the rats with the central venous occlusion [10] or superior mesenteric artery occlusion [9], would be rapidly developed in the rats with superior mesenteric vein irremovable occlusion. Thereby, vice versa, from the viewpoint of the BPC 157 therapy, the rapid upgrading of venous system function may be an essential common point to prevent and reverse the noxious chain of events and attenuate all harmful consequences [6,7,8,9,10]. Additionally, a life-threatening syndrome that would occur rapidly in the rats with the artificially maintained complete superior mesenteric vein occlusion, needs to be also counteracted rapidly. These may be the portal and inferior caval vein hypertension, abdominal aorta hypotension, and centrally, hypertension in the superior sagittal sinus (and thereby, rapid brain swelling), ECG disturbances, progressing venous and arterial thrombosis, and the multiple organs lesions, heart, lung, liver, kidney and gastrointestinal tract, in particular, as well as brain.

Finally, for the superior mesenteric injury therapy, and its mechanism investigated from a number of perspectives [1,2,11], this appears to be the novel combined point for further pathology evaluation (i.e., the entire noxious syndrome, in particular, vascular failure, and activation of the collateral pathway as a rescue) and the therapy effect as an indicative proof-of-concept.

## 2. Materials and Methods

### 2.1. Animals

This study was conducted with 12 weeks old, 200 g body weight, male albino Wistar rats, randomly assigned at 6 rats/group/interval. Rats were bred in-house at the Pharmacology Animal Facility, School of Medicine, Zagreb, Croatia. The animal facility was registered by the Directorate of Veterinary (Reg. No: HR-POK-007). Laboratory rats were acclimated for five days and randomly assigned to their respective treatment groups. Laboratory animals were housed in polycarbonate (PC) cages under conventional laboratory conditions at 20–24 °C, relative humidity of 40–70% and noise level 60 dB. Each cage was identified with dates, number of study, group, dose, number and sex of each animal. Fluorescent lighting provided illumination 12 h per day. Standard good laboratory practice (GLP) diet and fresh water was provided ad libitum. Animal care was in compliance with standard operating procedures (SOPs) of the Pharmacology Animal Facility, and the European Convention for the Protection of Vertebrate Animals used for Experimental and other Scientific Purposes (ETS 123).

This study was approved by the local Ethic Committee. Ethical principles of the study complied with the European Directive 010/63/E, the Law on Amendments to the Animal Protection Act (Official Gazette 37/13), the Animal Protection Act (Official Gazette 135/06), the Ordinance on the protection of animals used for scientific purposes (Official Gazette 55/13), Federation of European Laboratory Animal Science Associations (FELASA) recommendations and the recommendations of the Ethics Committee of the School of Medicine, University of Zagreb. The experiments were assessed by observers blinded as to the treatment.

### 2.2. Drugs

Medication was administered as described previously [6,7,8,9,10], without use of a carrier or peptidase inhibitor, for stable gastric pentadecapeptide BPC 157, a partial sequence of the human gastric juice protein BPC, which was freely soluble in water at pH 7.0 and in saline. BPC 157 (GEPPPGKPADDAGLV, molecular weight 1419; Diagen, Ljubljana, Slovenia) was prepared as a peptide with 99% high-performance liquid chromatography (HPLC) purity, with 1-des-Gly peptide being the main impurity. The dose and application regimens were as described previously [6,7,8,9,10].

### 2.3. Experimental Protocol

The described protocol used in the peripheral and central vascular occlusion syndromes [9,10] was consistently used also in the present study.

Briefly, in deeply anesthetized rats (intraperitoneal (ip) injected 40 mg/kg thiopental (Rotexmedica, Trittau, Germany) and 10 mg/kg diazepam (Apaurin; Krka, Novo Mesto, Slovenia)), we made complete occlusion of the end of the superior mesenteric vein (ligation) just below joining of the lienal vein. Thereby, permanent occlusion by ligation of the superior mesenteric vein leads to permanent alteration of blood flow, and continuously progressing course.

For all of the rats with ligation of the superior mesenteric vein, sacrificed at 30 min ligation time, medication was at 1 min ligation time, 10 µg/kg BPC 157, 10 ng/kg BPC 157, or 5 mL/kg saline. It was given intraperitoneally, as 1 mL/rat abdominal bath.

For venography, medication (10 µg/kg BPC 157, 10 ng/kg BPC 157 or 5 mL/kg saline) was applied intraperitoneally, as 1 mL/rat abdominal bath, at 15 min ligation time, just before venography.

Recording of the brain swelling was performed in rats at 15 min after the complete calvariectomy was performed. Briefly, six burr holes were drilled in three horizontal lines, all of them medially to the superior temporal lines and temporalis muscle attachments. The rostral two burr holes were placed just basal from the posterior interocular line, the basal two burr holes were placed just rostral to the lambdoid suture (and transverse sinuses) on both sides, respectively, and the middle two burr holes were placed in the line between the basal and rostral burr holes.

A laparotomy was made for the corresponding presentation of the peripheral veins (superior mesenteric, inferior mesenteric, inferior anterior pancreaticoduodenal, jejunal, middle colic, left colic, portal, inferior caval) and recording with a camera attached to a VMS-004 Discovery Deluxe USB microscope (Veho, Dayton, OH, USA) performed until the end of the experiment, and assessed at 5, 15, and 30 min ligation time.

### 2.4. Venography

Venography was performed in rats with a ligation of the superior mesenteric vein at 15 min post-ligation, using a C-VISION PLUS fluoroscopy unit (Shimadzu, Chiyoda, Tokyo, Japan) [6,7,8,9,10]. In total, 1 mL throughout 45 sec warmed Omnipaque 350 (iohexol) non-ionic contrast medium (GE Healthcare, Arlington Heights, IL, USA) was injected into the superior mesenteric vein below occlusion. The contrast medium was visualized under real-time to ensure adequate filling. A subtraction mode was used to record the images at 14 frames per second. At 15 min post-ligation, venograms were taken, captured, and digitized into files on a personal computer and were analyzed using ISSA image software (ISSA Network Station Version 4.0., Vamstec, Zagreb, Croatia). Venography assessment includes rats having a full presentation of collaterals and bypassed occlusion.

### 2.5. Superior Sagittal Sinus, Portal, Superior Mesenteric and Caval Vein and Abdominal Aorta Pressure Recording

As described before [6,7,8,9,10], recordings were made in deeply anesthetized rats with a cannula (BD Neoflon™ Cannula) connected to a pressure transducer (78534C MONITOR/ TERMINAL; Hewlett Packard, Houston, TX, USA) inserted into the superior sagittal sinus portal vein, superior mesenteric vein and inferior vena cava, and abdominal aorta at the level of the bifurcation at 30 min post-ligation after five minutes recording. For superior sagittal sinus pressure recording, we made a single burr hole in the rostral part of the sagittal suture, above the superior sagittal sinus, and cannulated superior sagittal sinus anterior part by Braun intravenous cannulas, and then, we laparatomized rats to cannulate portal vein, superior mesenteric vein, inferior caval vein, and abdominal aorta, for portal vein, superior mesenteric vein, inferior caval vein and abdominal aorta pressure recording.

Notably, normal rats exhibited a superior sagittal sinus pressure −24–−27 mmHg, superior mesenteric pressure and portal pressure of 3–5 mmHg similar to that of the inferior vena cava, though with at least 1 mmHg higher values in the portal vein. By contrast, abdominal aorta blood pressure values were 100–120 mm Hg at the level of the bifurcation [6,7,8,9,10].

### 2.6. ECG Recording

ECGs were recorded continuously in deeply anesthetized rats for all three main leads, by positioning stainless steel electrodes on all four limbs using an ECG monitor with a 2090 programmer (Medtronic, Minneapolis, MN, USA) connected to a Waverunner LT342 digital oscilloscope (LeCroy, Chestnut Ridge, NY, USA) at 30 min ligation time. This arrangement enabled precise recordings, measurements and analysis of ECG parameters [6,7,8,9,10].

### 2.7. Thrombus Assessment

On being euthanized, the superior sagittal sinus, and peripherally, the portal vein, inferior caval vein, superior mesenteric vein, lienal vein and superior mesenteric artery were removed from the rats, and clots were weighed [6,7,8,9,10].

### 2.8. Brain Volume and Vessels Presentation Proportional with the Change of the Brain or Vessels Surface Area

We used protocol previously described [9,10]. The presentation of the brain, and peripheral veins (superior mesenteric and inferior mesenteric, portal, inferior caval, inferior anterior pancreaticoduodenal, jejunal, middle colic, left colic and inferior caval) was recorded in deeply anaesthetized rats, with a camera attached to a VMS-004 Discovery Deluxe USB microscope (Veho, Dayton, OH, USA), before procedure in normal, and then, in rats with ligated superior mesenteric vein after procedure, before and after therapy as well as at the 5, 15 and 30 min ligation time before sacrifice. The border of the brain or veins in photograph was marked using ImageJ computer software and then, the surface area (in pixels) of the brain or veins was measured using a measuring function. This was done with brain photographs before the application and at intervals after the application for both control and treated animals. In the rats with occluded mesenteric vein, the brain or veins area before application was marked as 100% and the ratio of each subsequent brain area to the first area was calculated ($\frac{A_{2}}{A_{1}}$). Starting from square-cube law Equations (1) and (2) an equation for change of brain volume proportional with the change of the brain surface area (6) was derived. In expressions (1)–(5) l is defined as any arbitrary one dimensional length of brain (for example rostro-caudal length of the brain); used only for defining one dimensional proportion (l_2_/l_1_) between two observed brains and as an inter-factor (and because of that not measured [6]) for deriving final expression (6). The procedure was as follows: $A_{2}=A_{1}\times {\left(\frac{l_{2}}{l_{1}}\right)}^{2}$ (1) (square-cube law), $V_{2}=V_{1}\times {\left(\frac{l_{2}}{l_{1}}\right)}^{3}$ (2) (square-cube law), $\frac{A_{2}}{A_{1}}={\left(\frac{l_{2}}{l_{1}}\right)}^{2}$ (3) (from (1), after dividing both sides by A_1_),$\frac{l_{2}}{l_{1}}=\sqrt{\frac{A_{2}}{A_{1}}}$ (4) (from (3), after taking square root of both sides), $\frac{V_{2}}{V_{1}}={\left(\frac{l_{2}}{l_{1}}\right)}^{3}$ (5) (from (2), after dividing both sides by V_1_), $\frac{V_{2}}{V_{1}}={\left(\sqrt{\frac{A_{2}}{A_{1}}}\right)}^{3}$ (6) (qfter incorporating expression (4) into Equation (5)).

### 2.9. Stomach, Duodenum, Serosal Disturbances Presentation, Liver and Spleen Weights

The presentation of the gross lesions in gastrointestinal tract and serosal disturbances was recorded in deeply anaesthetized rats, with a camera attached to a VMS-004 Discovery Deluxe USB microscope (Veho, Dayton, OH, USA). At 30 min post-ligation, we assessed hemorrhagic congestive areas in the stomach and duodenum (sum of the longest diameters, mm). Serosal disturbances (hemorrhage, vessels ramification, arterial filling, congestion) were assessed in jejunum, cecum and ascending colon and scored 0–4, as follows. We scored the bleeding on the intestinal surface as follows: 0—no bleeding, 1—barely indicated bleeding (diameter of the hematoma on the intestinal surface <1 mm), 2—mild bleeding (diameter of the hematoma on the intestinal surface >1–2 mm), 3—moderate bleeding (diameter of the hematoma on the intestinal surface >2–4 mm), 4—intense bleeding (diameter of the hematoma on the intestinal surface >4 mm), 5—very intense bleeding (flowing bleeding), and venous congestion 0—venous congestion not present, 1—barely indicated venous congestion (vein thickness up to 0.5 mm), 2—mild venous congestion (vein thickness > 0.5–1 mm), 3—moderate venous congestion (vein thickness > 1–2 mm), 4—intense venous congestion (vein thickness > 2–2.5 mm), 5—massive venous congestion (vein thickness > 2.5 mm). Arterial filling was scored as follows: 0—arterial filling not noticeable, 1—barely indicated arterial filling (artery thickness up to 0.5 mm), 2—mild arterial filling (artery thickness > 0.5–0.75 mm), 3—moderate arterial filling (artery thickness > 0.75–2 mm), 4—intensive arterial filling (artery thickness > 2–2.5 mm), 5—massive arterial filling (artery thickness > 2.5 mm) as well as arterial ramification 0—no noticeable ramification, 1—barely indicated arterial ramification (visible 2 branches), 2—mild arterial ramification (visible 3 branches), 3—moderate arterial ramification (visible 4 branches), 4—intensive arterial ramification (visible 5 branches), 5—massive arterial ramification (visible 6 or more branches).

Liver and spleen weights were expressed as a percent of the total body weight (for normal rats, liver 3.2–4.0% and spleen 0.20–0.26%).

### 2.10. Bilirubin and Enzyme Activity

To determine the serum levels of aspartate transaminase (AST), alanine transaminase (ALT, IU/L), and total bilirubin (µmol/L), blood samples were collected immediately after euthanasia and were centrifuged for 15 min at 3000 rpm. All tests were performed using an Olympus AU2700 analyzer with original test reagents (Olympus Diagnostics, Southend-on-Sea, UK & Ireland) [15]. However, since there was no increase in bilirubin, the data were not shown.

### 2.11. Microscopy

Tissue specimens from brain, liver, kidney, spleen, stomach, duodenum, jejunum, ascending colon, lungs and heart were obtained from rats with superior mesenteric vein ligation at 30 min ligation time. These were fixed in buffered formalin (pH 7.4), for 24 h, dehydrated, and embedded in paraffin wax. The samples were stained with hematoxylin-eosin. Tissue injury was evaluated microscopically by a blinded examiner. Specifically, the brains were dissected using coronal section with mandatory 2 sections according to NTP-7, Levels 3 and 6, due to neuroanatomic subsites present in certain brain sections [39]. At NTP-7 Level 3 we observed area of fronto-parietal cortex, hippocampus, thalamus and, hypothalamus. At NTP-7 Level 6 we analyzed cerebellar cortex morphology. Brain coronal blocks were embedded in paraffin, sectioned at 4 μm, stained with H&E and evaluated by light microscopy using neuropathological scoring.

#### 2.11.1. Brain Histology

Brain injury in different regions was evaluated using a semiquantitative neuropathological scoring system as described [40] (Table 1), providing a common score 0–8, grade 0 indicates no histopathologic damage.

#### 2.11.2. Lung Histology

The following scoring system to grade the degree of lung injury was used in lung tissue analysis. Features were focal thickening of the alveolar membranes, congestion, pulmonary edema, intra-alveolar hemorrhage, interstitial neutrophil infiltration, and intra-alveolar neutrophil infiltration. Each feature was assigned a score from 0 to 3 based on its absence (0) or presence to a mild (1), moderate (2), or severe (3) degree, and a final histology score was determined [41].

#### 2.11.3. Renal, Liver, Heart Histology

The criteria for renal injury was based on degeneration of Bowman space and glomeruli, degeneration of proximal and distal tubule, vascular congestion and interstitial edema. The criteria for liver injury were vacuolization of hepatocytes and pyknotic hepatocyte nuclei, activation of Kupffer cells and enlargement of sinusoids. Each specimen was score using a scale ranging from 0 to 3 (0: none, 1: mild, 2: moderate, and 3: severe) for each criterion, and a final histology score was determined [42].

The hearth lesion estimation was based with dilatation and congestion of blood vessels within myocardium and coronary arteries as present (scored 1) or not present (scored 0).

#### 2.11.4. Intestinal Histology

A histologic scoring scale adapted from Chui et al. [43] was used for tissue scoring on a scale of 0–5 (normal to severe) in three categories (mucosal injury, inflammation, hyperemia/hemorrhage) for a total score of 0–15 as described by Lane et al. [44]. Morphologic features of mucosal injury were based on different grades of epithelia lifting, villi denudation and necrosis; grades of inflammation were graded from focal to diffuse according to lamina propria infiltration or subendothelial infiltration; hyperemia/hemorrhage graded from focal to diffuse according to lamina propria or subendothelial localization.

### 2.12. Oxidative Stress

At the end of the experiment (at 30 min) of ligation time, oxidative stress in the collected tissue samples (plasma, ICV) was assessed by quantifying thiobarbituric acid-reactive species (TBARS) as malondialdehyde (MDA) [32,33,34]. The tissue samples were homogenized in PBS (pH 7.4) containing 0.1 mM butylated hydroxytoluene (BHT) (TissueRuptor, Qiagen, Valencia, CA, USA) and sonicated for 30 s in an ice bath (Ultrasonic bath, Branson, MO, USA). Trichloroacetic acid (TCA, 10%) was added to the homogenate, the mixture was centrifuged at 3000 rpm for 5 min, and the supernatant was collected. Then, 1% TBA was added, and the samples were boiled (95 °C, 60 min). The tubes were then kept on ice for 10 min. Following centrifugation (14,000 rpm, 10 min), the absorbance of the mixture at the wavelength of 532 nm was determined.

The concentration of MDA was read from a standard calibration curve plotted using 1,1,3,3′ tetraethoxy propane (TEP). The extent of lipid peroxidation was expressed as MDA using a molar extinction coefficient for MDA of 1.56 × 105 mol/L/cm. The protein concentration was determined using a commercial kit. The results are expressed in nmol/g of protein.

### 2.13. Statistical Analysis

Statistical analysis was performed by parametric one-way analysis of variance (ANOVA), with post-hoc Newman–Keuls test and non-parametric Kruskal–Wallis test and subsequently the Mann–Whitney U test to compare groups. Values were presented as the mean ± standard deviation (SD) and as the minimum/median/maximum. To compare the frequency difference between groups, the chi-square test or Fischer’s exact test was used. *p* < 0.05 was considered statistically significant.

## 3. Results

We revealed the stable gastric pentadecapeptide BPC 157 as the effective therapy against the irremovable occlusion of the superior mesenteric vein, and consequent syndrome. The perilous syndrome occurred centrally and peripherally (blood pressure disturbances (intracranial (superior sagittal sinus) hypertension, portal and caval hypertension, and aortal hypotension), thrombosis, ECG disturbances). Peripherally, there were constant vein congestion and failure (portal vein, superior mesenteric vein, inferior caval vein, inferior anterior pancreaticoduodenal vein, jejunal vein, inferior mesenteric vein, middle colic vein, left colic vein), failed presentation of the bypassing loops (venography, gross assessment), gastrointestinal lesions and other organs lesions, heart, lung, liver, kidney and increased liver and spleen weight. Centrally, there were brain swelling, increased brain volume and brain lesions in all four investigated areas, cortex, hippocampus, hypothalamus and thalamus. MDA levels was a confirmative result of damaged intestinal mucosal integrity. All these disturbances (including increased enzymes serum values were counteracted by the application of the stable gastric pentadecapeptide BPC 157.

### 3.1. Perilous Syndrome Occurred Centrally and Peripherally

#### 3.1.1. Portal, Superior Mesenteric and Caval Vein and Abdominal Aorta and Superior Sagittal Sinus Pressure Recording

**Figure 1:**
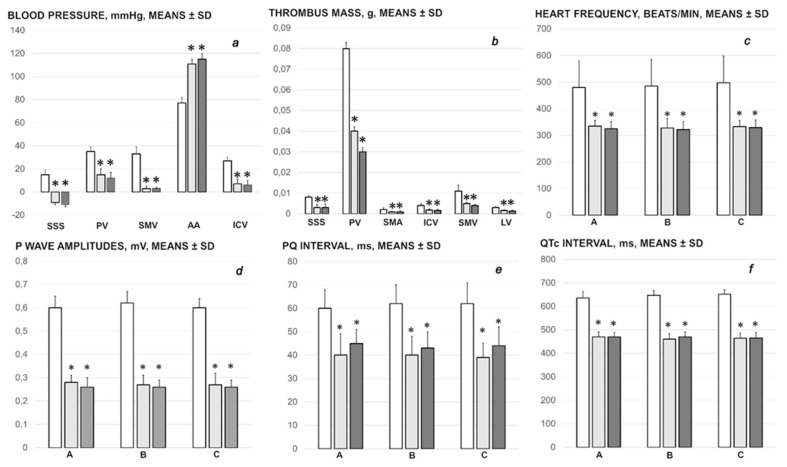
Blood pressure (**a**) and thrombus (**b**) presentation in the rats with the irremovable occlusion of the superior mesenteric vein (superior sagittal sinus (SSS), portal vein (PV), abdominal aorta (AA), inferior caval vein (ICV), superior mesenteric vein (SMV) and lienal vein (LV)). Assessment at the end of the 30 min ligation period. BPC 157 10 µg/kg (light gray bars), 10 ng/kg (dark gray bars); saline 5 mL/kg (white bars) given intraperitoneally at 1 min ligation time. ECG changes (**c**–**f**) at 5 min (A), 15 min (B) and 30 min (C) ligation time. Six rats/group/interval. Means ± SD, * *p* ˂ 0.05, at least, vs. control.

Without therapy, the rats with the occluded end of the superior mesenteric vein continuously exhibited the severe intracranial, portal, superior mesenteric and caval hypertension (portal hypertension exceeding caval hypertension), and aortal hypotension. The occlusion of the end of the superior mesenteric vein (ligation) immediately substituted the normal (negative) pressure in the superior sagittal sinus with the increased (positive) pressure (Figure 1). With BPC 157 therapy, µg- and ng-regimens, the severe portal, superior mesenteric and caval hypertension and aortal hypotension were rapidly attenuated or resolved. The increased (positive) pressure in the superior sagittal sinus was immediately substituted with the negative pressure.

#### 3.1.2. Thrombosis

In the rats with occluded superior mesenteric vein, thrombosis rapidly appeared peripherally and centrally. The largest clot appeared in the portal vein, and then in the inferior caval vein, superior mesenteric vein, lienal vein and superior mesenteric artery, as well as centrally, in the superior sagittal sinus (Figure 1). BPC 157, given at 1 min ligation time, markedly counteracted and reversed thrombosis presentation.

#### 3.1.3. ECG Recording

Regularly, ECG recordings showed severe tachycardia and peaked P waves, prolonged PQ and QTc intervals, which were markedly counteracted by BPC 157 regimens (Figure 1). Likewise, control rats with the occluded superior mesenteric vein presented consistent ST-elevation (0.5 ± 0.1, means ± SD) throughout the experiment, which was absent in BPC 157 treated rats (*p* ˂ 0.05, at least).

### 3.2. Perilous Syndrome Occurred Peripherally

#### 3.2.1. Vein Congestion

**Figure 2:**
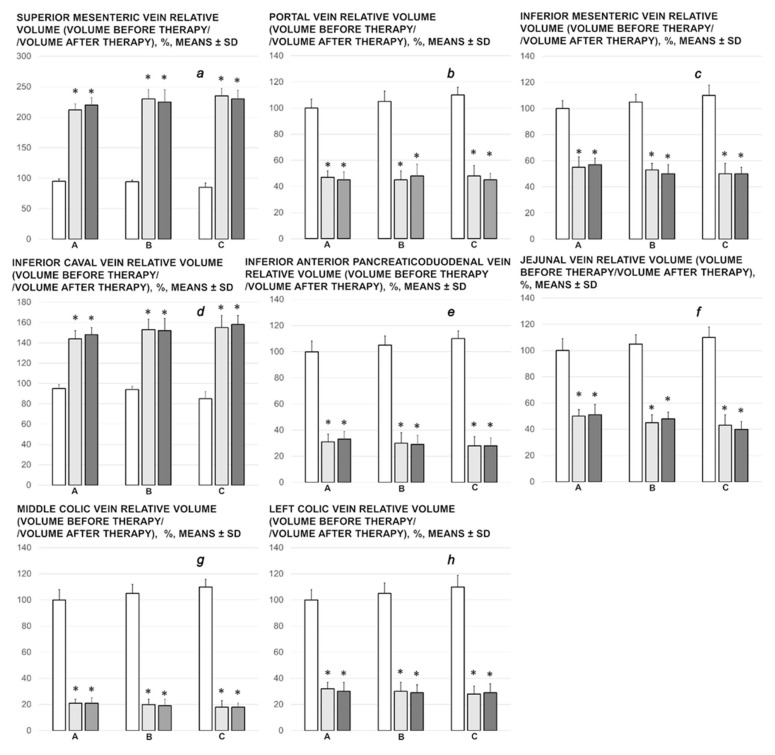
Relative volume of the veins (**a**–**h**) (superior mesenteric vein (**a**), portal (**b**), inferior mesenteric vein (**c**), inferior caval vein (**d**), inferior anterior pancreaticoduodenal vein (**e**), jejunal (**f**), middle colic vein (**g**), and left colic vein (**h**)), volume before therapy/volume after therapy, %, in the rats with ligated superior mesenteric vein, at 5 min (A), 15 min (B) and 30 min (C) ligation time. BPC 157 10 µg/kg (light gray bars), 10 ng/kg (dark gray bars); saline 5 mL/kg (white bars) given intraperitoneally at 1 min ligation time. Six rats/group/interval. Means ± SD, * *p* ˂ 0.05, at least, vs. control.

**Figure 3:**
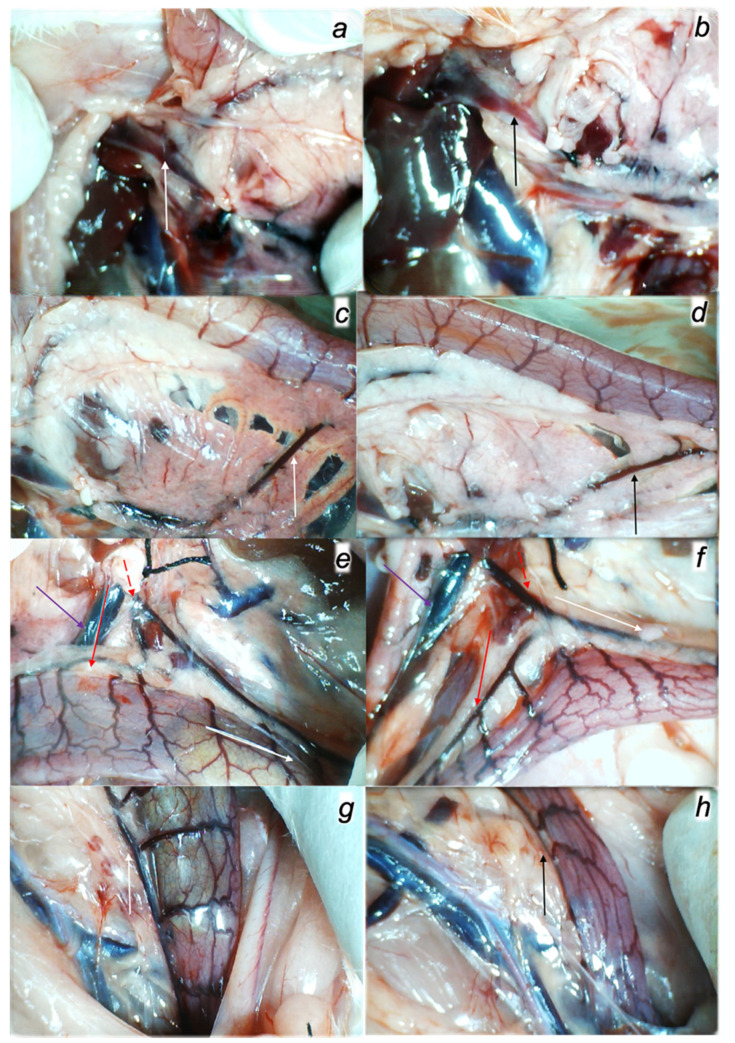
Collateral pathways gross presentation in rats with the occluded end of the superior mesenteric vein in the rats that received BPC 157 therapy (right) or saline medication (left). Portal vein presentation upon inflow of the pyloric vein, congested in controls (**a**) (white arrow) and functioning in BPC 157-treated rats (**b**) (black arrow). Inferior anterior pancreaticoduodenal vein presentation (**c**,**d**), congested in controls (**c**), and functioning in BPC 157-treated rats (**d**) duodenum presentation and duodenal arcade vessels presentation as follow up to the reestablished communication with the superior anterior pancreaticoduodenal vein (and thereby superior mesenteric vein and portal vein communication in BPC 157 rats (**d**)), or vessels failure in controls (**c**). (**e**,**f**) Presentation of the middle colic vein (full read arrow), inferior mesenteric vein (dashed read arrow), left colic vein and arcade vessels (white full arrow) and superior mesenteric vein (violet arrow) in the control rats (**e**) and in the BPC 157-rats (**f**), as the alternative pathway toward inferior caval vein. (**g**,**h**) Rectal veins presentation as tortuous veins presentation in controls (**g**) (white arrow) and counteraction in BPC 157 rats (**h**) (black arrow).

Proportional change of the vein area was used for the assessment of the peripheral vessel failure development recording (Figure 2 and Figure 3).

This effect is parallel with the effect on the peripheral blood vessels failure (control rats) or peripheral vessels failure recovery (BPC 157). The rats with the ligated superior mesenteric vein rapidly develop peripheral vessels failure (proportional with the change of the vein surface area). Illustratively, superior mesenteric vein volume (volume before therapy) reveals an immediate increase to 180% over the healthy presentation.

Initial vein congestion induced with the irremovable occlusion of the superior mesenteric vein, without BPC 157 therapy, in the control rats that received saline, remained constant in all of the assessed intervals (5, 15, and 30 min ligation time) and veins, portal vein, superior mesenteric vein, inferior caval vein, inferior anterior pancreaticoduodenal vein, jejunal vein, inferior mesenteric vein, middle colic vein, and left colic vein. All of these veins regularly maintained the similar values like those before (saline) therapy.

With BPC 157 therapy, as vein pathway running over the end of the superior mesenteric vein occlusion (i.e., inferior anterior pancreaticoduodenal vein), or as an alternative pathway toward inferior caval vein (i.e., inferior mesenteric vein, the middle and left colic veins), the veins presentation was reversed in a particular way. Along with BPC 157 application, illustrative is the reversal of the congested vessel to the non-congested vessel with blood flow passing (close to healthy presentation) (superior mesenteric vein, inferior caval vein, volume before therapy > volume after therapy). Again, vessels are functioning, i.e., portal vein, inferior anterior pancreaticoduodenal vein, jejunal vein, inferior mesenteric vein, the middle and left colic veins (and thereby, volume before therapy < volume after therapy). These may provide the particularly activated pathways. The first pathway, superior mesenteric vein–inferior anterior pancreaticoduodenal vein–superior anterior pancreaticoduodenal vein–pyloric vein–portal vein pathway, illustrates directly reestablished superior mesenteric vein (decongested) and portal vein (refilled) connection and reestablished blood flow. Of note, the reversal of the failed function of the additional pathway to ascertain alternative bypassing blood flow toward inferior caval vein that would appear via the inferior mesenteric vein united with the middle colic vein, throughout its left colic vein (all refilled), illustrate the tortuous rectal veins presentation in the controls, which was fully counteracted in BPC 157 rats. Both are the presentation of the reorganized blood flow to compensate and bypass occluded end of the superior mesenteric vein, as seen at 5, 15 and 30 min ligation time.

#### 3.2.2. Venography in the Superior Mesenteric Vein

**Figure 4:**
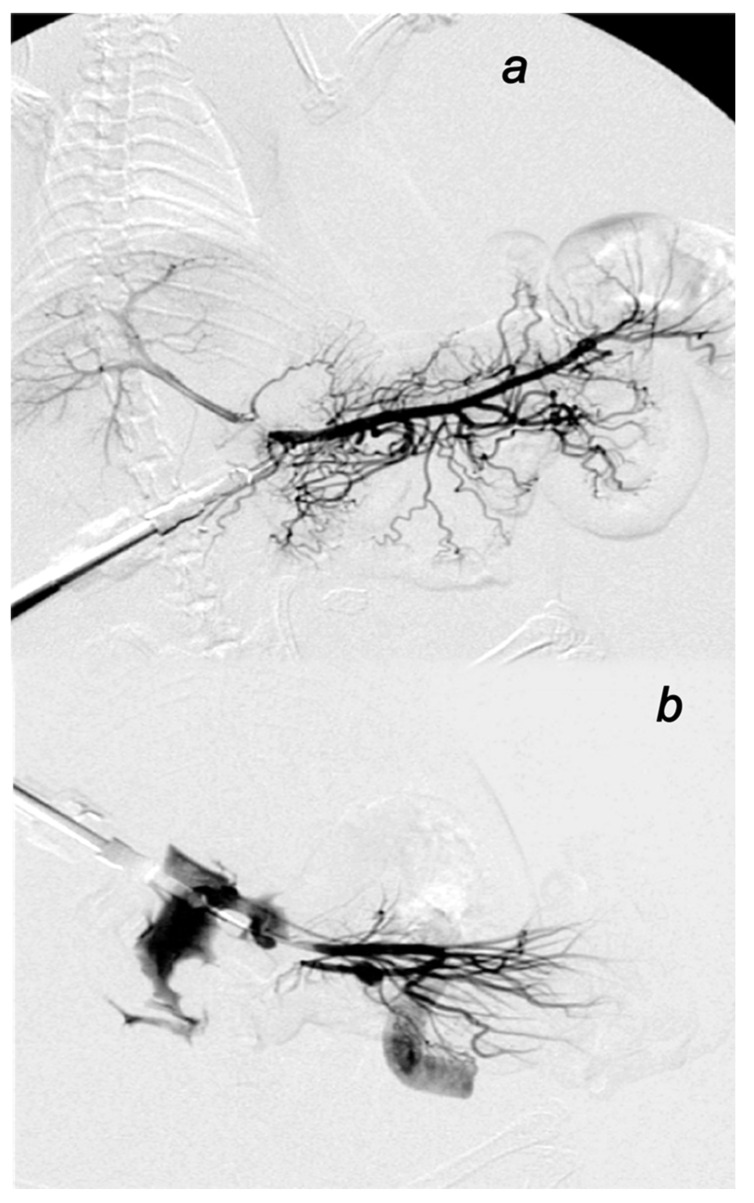
Venography. BPC 157 medication (**a**). After ligation of superior mesenteric vein, clear visualization of collaterals bypassing defect of superior mesenteric vein. Venography clearly showed the superior mesenteric vein–inferior anterior pancreaticoduodenal vein–superior anterior pancreaticoduodenal vein–pyloric vein–portal vein pathway, reestablished superior mesenteric vein and portal vein connection and reestablished blood flow, the mesenterico-portal confluent and bypassing defect of superior mesenteric vein. Contrast flow into main portal trunk, portal bifurcation on the left and right portal vein and intraparenchymal portal branches on the porto-venous (or parenchymal) liver phase. Without medication, control rats (**b**) with the ligated superior mesenteric vein regularly show poor presentation in the venography (1 mL through 30 sec in the superior mesenteric vein). Commonly, they respond with the rapid rupture of the superior mesenteric vein and lack of the activated collaterals.

Without medication, rats with the ligated superior mesenteric vein regularly show poor presentation in the venography (1 mL through 30 sec in the superior mesenteric vein) (Figure 4). Commonly, they respond with the rapid rupture of the superior mesenteric vein and lack of the activated collaterals.

BPC 157 medication fully counteracted these disturbances (*p* ˂ 0.05, at least, vs. control). Consistent with the evidenced counteraction of the portal, superior mesenteric and caval hypertension and the counteraction of the increased pressure in the superior sagittal sinus, superior mesenteric vein venography in the all BPC 157 rats revealed activated collaterals presentation and revealed bypassing of the termination of the superior mesenteric vein. Venography clearly showed the superior mesenteric vein–inferior anterior pancreaticoduodenal vein–superior anterior pancreaticoduodenal vein–pyloric vein–portal vein pathway, reestablished superior mesenteric vein and portal vein connection and reestablished blood flow. Presented were the portal and hepatic veins, and all superior mesenteric vein tributaries until the cecum veins. BPC 157 venography goes without congestion within hepatic veins with parenchymal liver phase.

Besides, with respect to the time of the application (i.e., 15 min ligation time), these findings indicated the reversal of the already advanced deleterious course.

#### 3.2.3. Gastrointestinal Lesions

**Figure 5:**
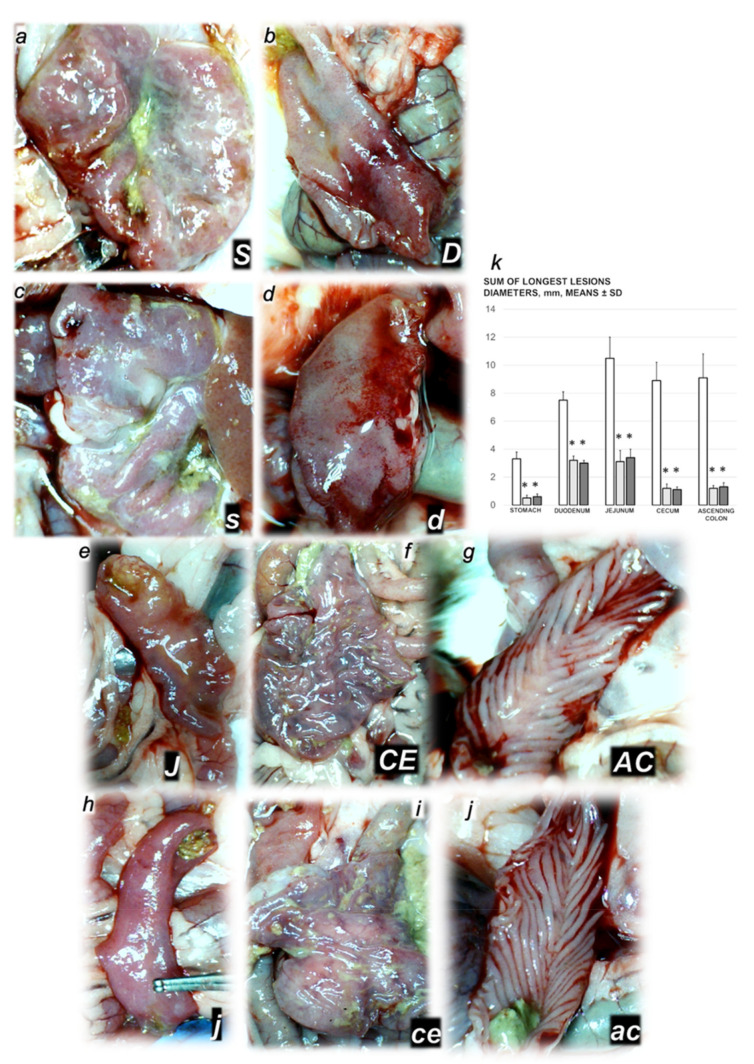
Illustrative gross gastrointestinal lesions presentation (**a**–**j,** black letters, lesions specifically indicated with white letters, small letters (control), capitals (BPC 157) as follows: stomach (**S**,**s**) (**a**,**c**) (**S** (BPC 157 (**a**), **s** (control (**b**)); duodenum (**D**,**d**) (**b**,**d**) (**D** (BPC 157 (**b**), **d** (control (**d**)); jejunum (**e**,**h**) (**J** (BPC 157) (**e**), **j** (controls) (**h**)); cecum (**CE** (BPC 157) (**f**), ce (controls) (**i**)); ascending colon (**AC** (BPC 157) (**g**), **ac** (controls) (**j**)). Lesion progression (**c**,**d**,**h**–**j**) or more preserved mucosa (**a**,**b**,**e**–**g**) in the gastrointestinal tract of the rats with the occluded end of the superior mesenteric vein, at 30 min after they had received saline therapy (small letters) or BPC 157 therapy (capitals). (**k**) Mucosal gastrointestinal lesions (sum of the longest lesions diameters, mm, means ± SD) at the end of the 30 min period following medication (BPC 157 10 µg/kg (light gray bars), 10 ng/kg (dark gray bars); saline 5 mL/kg (white bars)) given intraperitoneally. Six rats/group/interval. * *p* ˂ 0.05, at least, vs. control.

**Figure 6:**
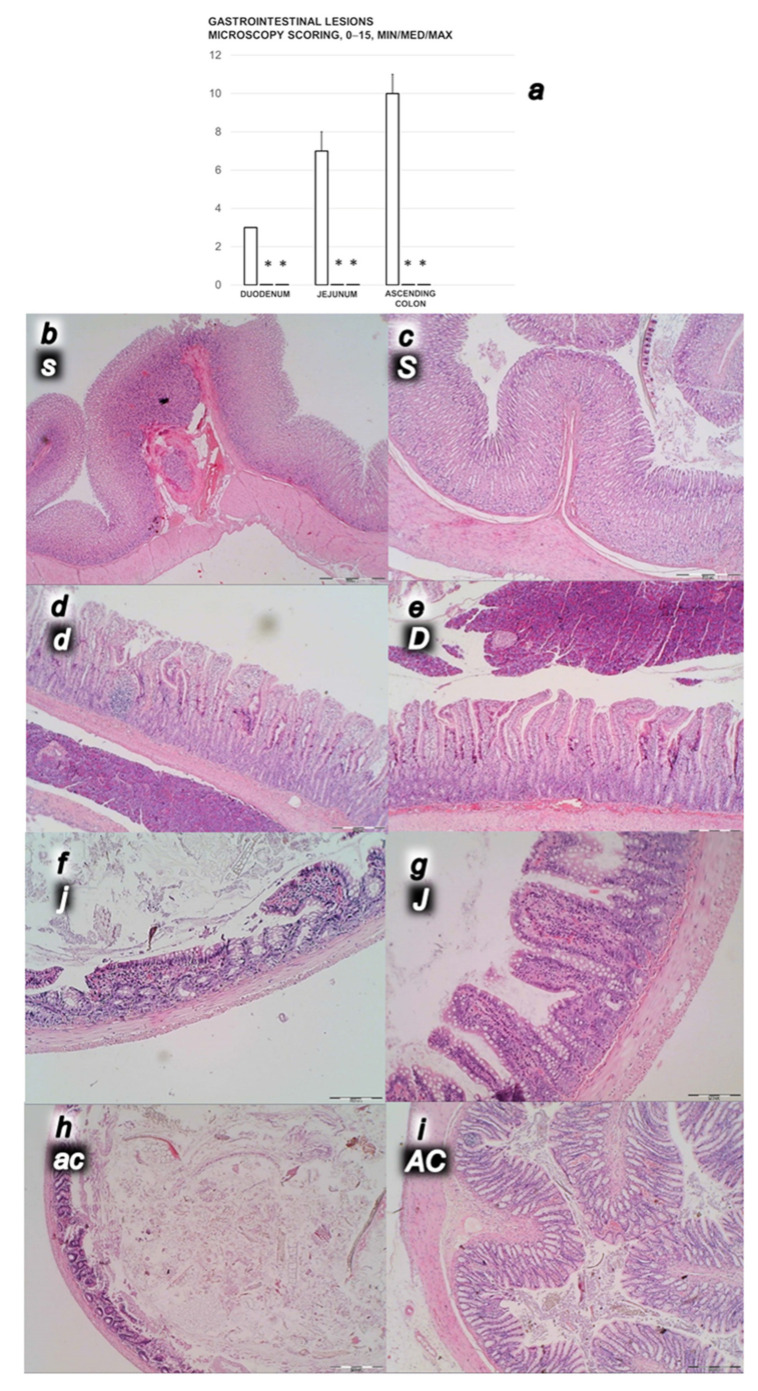
(**a**) Microscopy scoring of the lesions of the duodenum, jejunum and ascending colon (scored 0–15) (**a**) in the rats with ligated superior mesenteric vein at the end of the 30 min period following medication (BPC 157 10 µg/kg (light gray bars), 10 ng/kg (dark gray bars); saline 5 mL/kg (white bars)) given intraperitoneally. Six rats/group/interval. * *p* ˂ 0.05, at least vs. control. (**b**–**i**) Characteristic microscopy presentation of the affected gastrointestinal tract (**b**–**i**, black letters, lesions specifically indicated with white letters, small letters (control), capitals (BPC 157), as follows: stomach (**s**,**S**), duodenum (**d**,**D**), jejunum (**j**,**J**), and ascending colon (**ac**,**AC**)). Note from stomach (**b**,**c**) (hyperemia in controls (**b**), unlike BPC 157 (**c**) rats), duodenum (**d**,**e**), (blunt villi with discrete villi reduction and cryptal hyperplasia in controls (**d**), unlike BPC 157 rats (**e**)), jejunum (**f**,**g**) (hyperemia and reduction of villi in controls (**f**), unlike BPC 157 rats (**g**)) and ascending colon (**h**,**i**) (dilatation of lumen, reduction of crypts, hyperemia of lamina propria in controls (**h**), unlike BPC 157 rats (**i**)). (HE; magnification ×20, scale bar 500 µm (**b**,**c**); ×40, scale bar 200 µm (**d**–**i**)).

**Figure 7:**
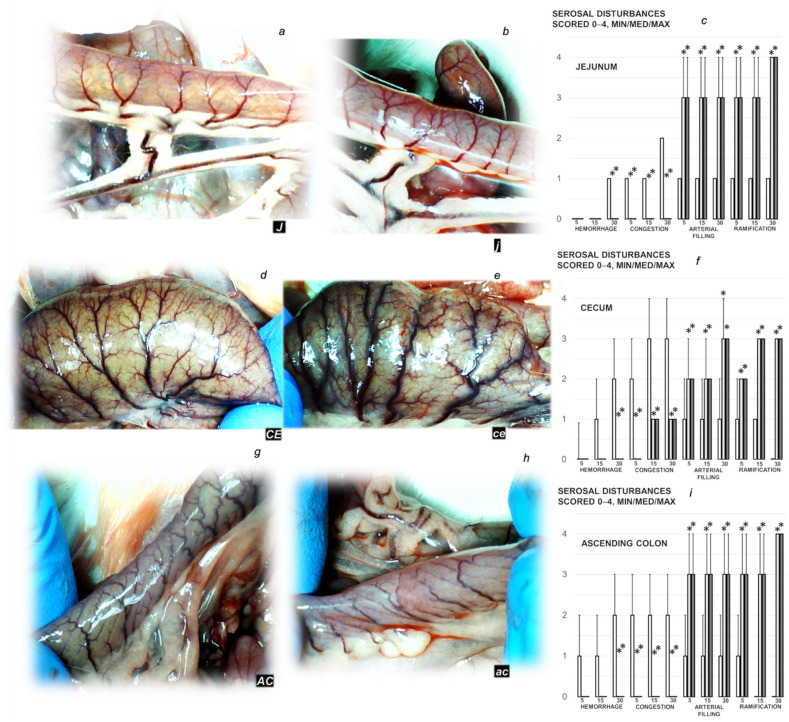
Serosal disturbances presentation (black letters **a**–**i**). Serosal blood vessels gross presentation in the rats with the occluded end of the superior mesenteric vein in the rats that received BPC 157 therapy (**a**,**d**,**g**) or saline medication (**b**,**e**,**h**). Presentation in the jejunum (**J** (BPC 157) (**a**), **j** (controls) (**b**)), cecum (**CE** (BPC 157) (**d**), **ce** (controls) (**e**)) and ascending colon (**AC** (BPC 157) (**g**), **ac** (controls) (**e**)). Increased ramification and no congestion in BPC 157 treated rats, unlike advanced congestion and poor ramification in corresponding controls. Serosal disturbances (hemorrhage, congestion, arterial filling, ramification, scored 0–4, Min/Med/Max) assessment in the jejunum (**c**), cecum (**f**) and ascending colon (**i**) in the rats with ligated superior mesenteric vein at the end of the 30 min period following medication (BPC 157 10 µg/kg (light gray bars), 10 ng/kg (dark gray bars); saline 5 mL/kg (white bars)) given intraperitoneally. Six rats/group/interval. * *p* ˂ 0.05, at least, vs. control.

Rats with ligated superior mesenteric vein regularly showed the severe lesions in the gastrointestinal tract (Figure 5, Figure 6 and Figure 7). Regularly, throughout the gastrointestinal tract, there were bleeding mucosal lesions, as well as at the serosa, the rats with ligated superior mesenteric vein presented the considerable hemorrhage and congestion, failed arterial filling and lack of ramification. Contrarily, BPC 157 rats presented markedly less mucosal lesions, and at the serosa considerably less hemorrhage and congestion, preserved arterial filling and advanced ramification seen as small collaterals rapidly presented and fully perfused interconnections between the neighboring vessels.

Controls with the occluded superior mesenteric vein showed marked transmural congestion within stomach, duodenum, small and large bowel wall, with an ascending sequence from the proximal to the distal part of the gastrointestinal tract. Illustratively, in the proximal parts of the gastrointestinal tract, there were only dilated capillaries in the lamina propria. Within small and large bowel mucosa, focal hemorrhage appeared in the lamina propria. Mild mucosal injury appeared with blunt duodenal villi and mild hyperplasia of the crypts; more severe mucosal injury with reduction of intestinal villi; even more severe mucosal injury with lumen dilatation of the colon and reduction of crypts. Contrarily, a marked lesions counteraction appeared with BPC 157 therapy. In the gastrointestinal tract of the rats with the occluded superior mesenteric vein that received BPC 157, all these changes were not found (total score 0)).

#### 3.2.4. Heart, Lung, Liver, Kidney Lesions

**Figure 8:**
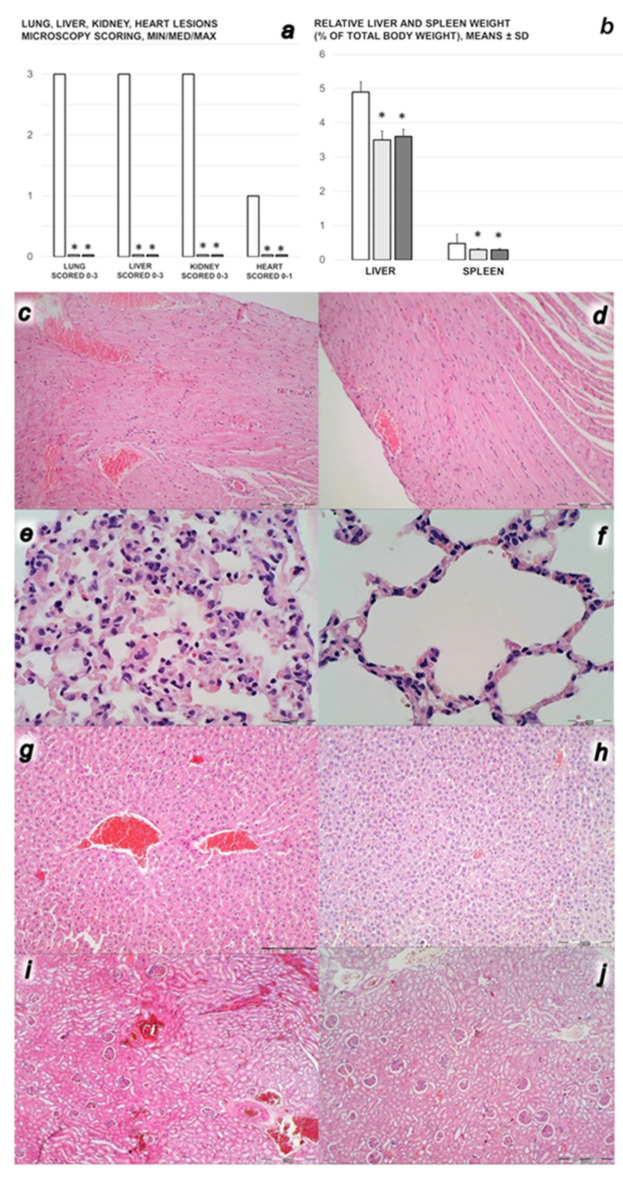
(**a**,**b**) Microscopy scoring of the lesions of the lung, liver, kidney (scored 0–3) and heart (scored 0–1), Min/Med/Max, (**a**), liver and spleen relative weight (% of total body weight, means ± SD), (**b**), in the rats with ligated superior mesenteric vein at the end of the 30 min period following medication (BPC 157 10 µg/kg (light gray bars), 10 ng/kg (dark gray bars); saline 5 mL/kg (white bars)) given intraperitoneally. Six rats/group/interval. * *p* ˂ 0.05, at least, vs. control. **c**–**j**. Characteristic microscopy presentation of the affected organs (**c**–**j**), heart (**c**,**d**), marked congestion within myocardium and large coronary branches in controls (**c**), unlike BPC 157 rats (**d**); lung (**e**,**f**), hyperemia and intraalveolar hemorrhage with margination of inflammatory cells in blood vessels in controls (**e**), unlike BPC 157 rats (**f**); liver (**g**,**h**), hyperemia in central veins and dilatation of sinusoids in controls (**g**), unlike BPC 157 rats (**h**); kidney (**i**,**j**), severe cortical and medullar hyperemia in controls (**i**), unlike BPC 157 rats (**j**). (HE; magnification x200, scale bar 50 µm (**c**,**d**); ×400, scale bar 20 µm (**e**–**f**)), magnification ×100, scale bar 100 µm (**g**–**j**).

In controls, lung congestion appeared with intralveolar hemorrhage, perivascular margination of neutrophils. In liver congestion, liver hyperemia in central veins and dilatation of sinusoid, pyknotic hepatocyte nuclei appeared. Additionally, kidney cortical and medullar hyperemia was found, and marked congestion in heart tissue, within myocardium and large coronary branches. In contrast, these changes were not present in the BPC 157 treated rats. In the spleen, BPC 157-treated rats have less apparent sinusoidal congestion, and dilatation and enlargement of red pulp leading to reduction of white pulp (Figure 8).

In addition, BPC 157 markedly attenuated the increased liver and spleen weight that otherwise would regularly appear in rats with the occluded superior mesenteric vein (Figure 8).

### 3.3. Perilous Syndrome Occurred Centrally

#### 3.3.1. Brain Swelling and Counteraction

**Figure 9:**
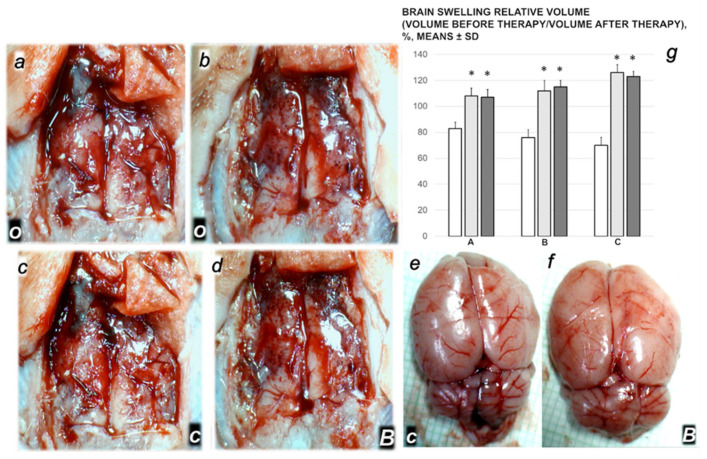
Brain swelling gross in vivo presentation in the rats with the occluded end of the superior mesenteric vein in the rats before the medication (**a**,**b**, black letters) (**o**, white letters). Further swelling progression (**c**, black letters) at 15 min after they had received saline therapy (**c**, white letters). Decreased swelling (**d**, black letters) at 15 min after they had received BPC 157 therapy (**B**, white letters). Swelling progression (**e**, black letters) at 30 min after they had received saline therapy (**c**, white letters). Decreased swelling (**f**, black letters) at 30 min after they had received BPC 157 therapy (**B**, white letters). Relative volume of the relative volume of brain swelling (**g**), volume before therapy/volume after therapy, %, in the rats with ligated superior mesenteric vein at 5 min (A), 15 min (B) and 30 min (C) ligation time. BPC 157 10 µg/kg (light gray bars), 10 ng/kg (dark gray bars); saline 5 mL/kg (white bars)) given intraperitoneally at 1 min ligation time. Six rats/group/interval. Means ± SD, * *p* ˂ 0.05, at least vs. control.

Proportional change of the vein or brain surface area was used for the assessment of the peripheral vessels failure development as well as brain-swelling recording (Figure 9).

The rats with the ligated superior mesenteric vein rapidly develop brain swelling (brain volume proportional with the change of the brain surface area reveals an immediate increase to the 120% over the healthy presentation).

As a consistent and prominent effect, BPC 157 therapy rapidly attenuates the brain swelling close to the normal, pre-procedure values, with µg- and ng-regimens.

#### 3.3.2. Brain Damage

**Figure 10:**
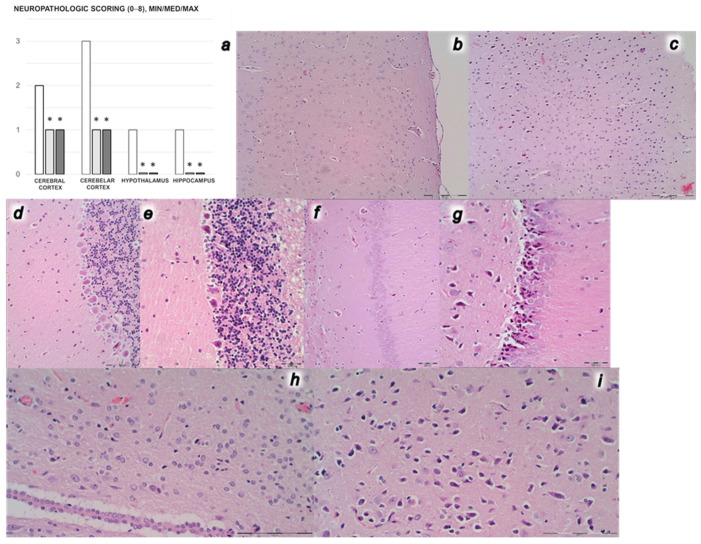
Microscopy scoring of the lesions of the brain lesions (**a**) (cerebral and cerebellar cortex, hypothalamus, hippocampus neuropathologic scoring (0–8)), Min/Med/Max, at the end of the 30 min period following medication (BPC 157 10 µg/kg (light gray bars), 10 ng/kg (dark gray bars); saline 5 mL/kg (white bars)) given intraperitoneally. Six rats/group/interval. * *p* ˂ 0.05, at least vs. control. Characteristic microscopy presentation of the affected brain (**b**–**i**). Cortex (**b**,**c**). Edema and hypoxia in controls (**c**), unlike BPC 157 rats (**b**). Cerebellum (**d**,**e**). Purkinje cells hypoxia in controls (**e**), unlike BPC 157 rats (**d**). Hippocampus (**f**,**g**). Hypoxia and red neurons (**g**), unlike BPC 157 rats (**f**). Hypothalamus (**h**,**i**). Hypoxia and red neurons in controls (**i**), unlike BPC 157 rats (**h**). (HE; magnification ×200 (**b**–**i**), scale bar 50 µm (**b**–**g**), scale bar 100 µm (**h**,**i**)).

Unlike rats that received BPC 157 medication and presented normal structure of cortex, controls presented significant lesions, in all four investigated regions, the cortex, hippocampus, hypothalamus and thalamus (Figure 10). Karyopyknosis was increased showing larger area with the increased number of karyopyknotic cells of all four regions, cerebral and cerebellar cortex, hypothalamus, thalamus, hippocampus cortex, hypothalamus/thalamus. Neuropathologic changes in cerebral cortex areas revealed increased edema and congestion. Especially, there were karyopyknosis and degeneration of Purkinje cells of the cerebellar cortex and marked karyopyknosis of pyramidal cells of the hippocampus.

### 3.4. Oxidative Stress

**Figure 11:**
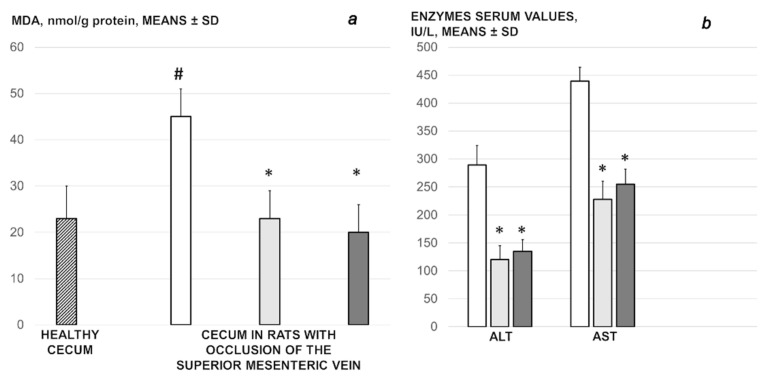
Oxidative stress (MDA, nmol/g protein, means ± SD) (**a**), enzyme serum values (IU/L, means ± SD) (**b**) in the rats with ligated superior mesenteric vein at the end of the 30 min period following medication (BPC 157 10 µg/kg (light gray bars), 10 ng/kg (dark gray bars); saline 5 mL/kg (white bars)) given intraperitoneally. Six rats/group/interval. * *p* ˂ 0.05, at least vs. control, # *p* ˂ 0.05, at least vs. healthy (dashed bar).

Without medication, rats with the ligated superior mesenteric vein regularly showed increased MDA values (Figure 11). This was completely counteracted in the rats that received BPC 157 medication.

### 3.5. Enzymes

Serum ALT and AST values increased in controls; they were lower in rats treated with BPC 157 (Figure 11).

In summary (see also data, Figure S1), the evidence was provided that in the rats with the ligated superior mesenteric vein BPC 157 therapy rapidly attenuated the severe portal and caval hypertension and aortal hypotension. That emerging of the rapid collateral vessels recruitment also rapidly eliminates the increased pressure in the superior sagittal sinus. Thus, there is rapid resolving adequately of the anatomical imbalance in venous drainage along with acting both peripherally and centrally. The brain, heart, lung, liver, kidney, gastrointestinal lesions, thrombosis attenuation appears as the automated result.

## 4. Discussion

As the novel demonstration with the stable gastric pentadecapeptide BPC 157 therapy, the injurious regimen with superior mesenteric vein occlusion is corresponding to the other vessel occlusion syndromes [6,7,8,9,10]. Thus, such rapid full presentation of the entire peripheral and central syndrome, irremovable throughout the 30 min period, consistently emphasized the given occlusion breakpoint (end of the superior mesenteric vein) as an additional particular occlusion point responsible for the following course, as shared common deleterious outcome noted with the particular vessels irremovable occlusion. Rapidity of the combined events thereby is consequent to an alike blood vessel function failure as induced by direct (occlusion) vessel obstruction. Likely, this means irremovable (endothelium) lesions; whatever the full peripheral and central syndrome would appear in addition to each other, due to the prime peripheral or central lesion, these lesions should appear as an essential cause–consequence vicious circle, multiorgan dysfunction syndrome. Conceptually, this may be either of brain swelling and intracranial (superior sagittal sinus) hypertension, brain lesions, portal and caval hypertension, aortal hypotension, widespread thrombosis in veins and arteries, congested superior mesenteric vein and inferior caval vein, ECG disturbances, heart, lung, liver kidney and gastrointestinal lesions. Thereby, the pleiotropic beneficial therapy BPC 157 effect, known to rapidly attenuate/eliminate the consequences of the irremovable occlusion of the various vessels, peripheral and central, and activate particular bypassing loops in relation to the given occlusion, may have considerable conceptual importance [6,7,8,9,10].

Here, considering all of the beneficial effects of the BPC157 administration [6,7,8,9,10], the significance of the specific starting point (venography (the rapid rupture of the superior mesenteric vein and lack of the activated collaterals occurred in controls); gross recording) is fully supported. The compensated essential superior mesenteric vein occlusion illustrates directly reestablished superior mesenteric vein and portal vein connection and reestablished blood flow via an activated vein pathway running over the end of the superior mesenteric vein occlusion (i.e., inferior anterior pancreaticoduodenal vein–superior anterior pancreaticoduodenal vein–pyloric vein pathway, which completely failed in controls). The reversal of the congested vessels (i.e., assessed were the portal vein, superior mesenteric vein, inferior caval vein, inferior anterior pancreaticoduodenal vein and jejunal vein) goes to their presentation as the non-congested vessels, with blood flow passing (close to normal presentation). As an additional more remote, specific bypassing pathway simultaneously activated, united with the middle colic vein, is the inferior mesenteric vein throughout its left colic branch and may fairly contribute to ascertain alternative bypassing blood flow achieved via inferior caval vein. Illustratively, the failed presentation of these vessels is reversed to the functioning presentation in the BPC 157 rats but remained failed in the controls. These findings are in line with the similar therapy effect in previous major vessel occlusion-syndromes [6,7,8,9,10].

Thus, in the rats with the irremovable occlusion of the superior mesenteric vein, the brain swelling and increased intracranial (superior sagittal sinus) hypertension may be both the cause and the consequence of the severe brain lesions, in all four investigated regions, the cortex, hippocampus, hypothalamus and thalamus. This may be, however, initiated either centrally or peripherally, providing the alike noxious course presentation in the rats with the occluded superior sagittal sinus [10] and in the rats with occluded superior mesenteric artery [9] as well as in the rats with occluded superior mesenteric vein. Thus, commonly, the rats with central venous occlusion [10], and the rats with superior mesenteric artery occlusion [10], as well as the rats with the irremovable occlusion of the superior mesenteric vein could not drain venous blood adequately for a given cerebral blood inflow without raising venous pressures, and thereby, suddenly goes such venous and intracranial hypertension [45,46,47,48,49].

Vice versa, BPC 157 therapy is in line with the presented normal structure in all four investigated brain regions of the rats with irremovable occlusion of the superior mesenteric vein. Superior sagittal sinus pressure is again within the normal negative values. Additionally, BPC 157 rapidly counteracted brain swelling. Together, these may suggest that the rats with BPC 157 therapy [6,7,8,9,10], even with major vessel (superior mesenteric vein) occlusion, could drain venous blood adequately for a given cerebral blood inflow without raising venous pressures, and thereby, may suddenly counteract such venous and intracranial hypertension. This may be likely since BPC 157 was shown to maintain normal (negative) pressure values even confronted with the additional challenges (volume application) originated within the cranium, or in the periphery, given cranial or peripheral intravenous challenge [10]. As such, along with its therapy effect in other vessel occlusion syndromes [6,7,8,9,10], this effect may be both the cause and the consequence of the no changes within myocardium, lung, liver and renal parenchyma. Additionally, both the cause and the consequence may be the counteracted ECG disturbances; eliminated portal and caval hypertension (note, BPC 157 may affect portal hypertension presentations whatever the cause, post-hepatic, hepatic and pre-hepatic [7,8,15,50]), and markedly attenuated aortal hypotension, and abrogated stomach hemorrhagic lesions, almost eliminated venous and arterial thrombosis, thereby counteracted stasis, as ascertained more adequate blood flow.

On the other hand, as a common pathway [6,7,8,9,10] may be the heart and lung as additional prime targets, noted in the peripheral and central vessel occlusion syndromes [6,7,8,9,10]; the rats with superior mesenteric vein occlusion, without therapy, with the severe brain injuries exhibited considerable lesions in the heart and in the lung. They exhibited peaked P wave, tachycardia, prolonged PQ and QTc intervals and ST-elevation, severe myocardial congestion, and congestion and hemorrhage in lung parenchyma, resembling acute respiratory distress syndrome exudative features. Then, all of these rats [6,7,8,9,10], and the rats with superior mesenteric vein occlusion especially, exhibited consequently the liver failure and kidney failure, progressing congestion, and extensive gastric hemorrhagic lesion, along with prominent portal and caval hypertension, inferior caval vein and superior mesenteric vein congestion. The escalating thrombosis is a shared common point of Virchow triad [6,7,8,9,10], reflecting a general stasis (i.e., large volume trapped in the damaged stomach, CNS and portal and caval vein tributaries may perpetuate the brain and heart ischemia as well), peripherally and centrally, and failed activation of the collateral bypassing pathways. It rapidly appeared in minute time, peripherally (i.e., the largest clot appeared in the portal vein, and then in the inferior caval vein, superior mesenteric vein, lienal vein and superior mesenteric artery), as well as centrally, in the superior sagittal sinus.

For the noted BPC 157 activity [6,7,8,9,10], its epithelium–endothelium cytoprotective effect [51] and specific “bypassing key” [6,7,8,9,10], also in the superior mesenteric vein occlusion, may be the conclusive evidence, the best defined by the observed effects themselves. A common point in the vascular occlusion studies could suggest the noted special interaction with NO-system and NO-agents in the various models and species [31]. BPC 157 induced the NO-release of its own [52,53], and counteracted induced hypertension and pro-thrombotic effect (L-NAME) [19,52], and induced hypotension and anti-thrombotic (L-arginine) effect [19,52]. The specific effects on blood pressure and thrombocytes function maintenance [17,18,19,52] and specific effect in vascular occlusion studies (always combined with the organ lesions antagonization, and counteracted blood pressure disturbances) [6,7,8,9,10] agree with a vasomotor tone carried out through BPC 157 specific activation of Src–Caveolin-1–endothelial nitric oxide synthase (eNOS) pathway [23], and maintenance of the prostaglandins system function [21,30]. Illustratively, BPC 157 counteracted the adverse effects of NSAIDs, COX-1 and COX-2 blockers [54,55,56,57,58,59] and might adjuvant arthritis in rats both preventing development and curing already established lesions [60]. Indomethacin cytoprotection studies [21] and mitigated leaky gut syndrome revealed the BPC 157 activity as stabilizer of cellular junction, via increasing tight junction protein ZO-1 expression, and transepithelial resistance [21]. There were inhibited mRNA of inflammatory mediators (iNOS, IL-6, IFNγ and TNF-α), increased expression of HSP 70 and 90, and antioxidant proteins, such as HO-1, NQO-1, glutathione reductase, glutathione peroxidase 2 and GST-pi [21]. Thus, these arguments against the damaging effect on vessel function can be also combined in the rats with superior mesenteric vein treated with BPC 157 with the reduced malondialdehyde (MDA), even to normal levels, as a confirmative result of both preserved and rescued intestinal mucosal integrity and vein integrity [9]. This occurs as before in both ischemic and reperfusion conditions in the various tissues (i.e., colon, duodenum, cecum, liver and veins) and plasma [6,7,9,13,14,15]. BPC 157 exhibited a specific effect on the *Egr*, *Nos*, *Srf*, *Vegfr*, *Akt1*, *Plcɣ*, and *Kras* pathways in the vessel that provides an alternative operating pathway (i.e., left ovarian vein as the key for the infrarenal occlusion-induced inferior caval vein syndrome in rats) [6]. Given in reperfusion in stroke-rats [20], BPC 157 therapy counteracted both early and delayed neural hippocampal damage. In hippocampal tissues, mRNA expression studies at 1 and 24 h, and strongly elevated (*Egr1*, *Akt1*, *Kras*, *Src*, *Foxo*, *Srf*, *Vegfr2*, *Nos3*, *Nos1*) and decreased (*Nos2*, *Nfkb*) gene expression (*Mapk1* not activated) may be a way how BPC 157 may act [20]. Counteraction of the retinal ischemia and severe damage induced by retrobulbar application of L-NAME may provide a possible important analogy [61].

Likely in the same way, along with encephalopathies [54,55,56,57,58,59], BPC 157 counteracts multiple pathologies in the gastrointestinal tract and liver [54,55,56,57,58,59]. Additionally, BPC 157 counteracts the various arrhythmias [62,63,64,65,66] (in particular, BPC 157 therapy normalizes the QTc duration in rats treated with neuroleptics, and prevents and recovers chronic heart failure [64,65]) and lung pathology (i.e., pulmonary hypertension syndrome in chicken [67], and intratracheal HCl instillation-induced lung lesions in rats [68]).

Finally, this study should overwhelm the general limiting point that animal studies per se may be cautious regarding their results. Here, although each of the many procedures used in the present study, if taken separately, may be probably seen from different perspectives, but taken together, all of these procedures prove each other and provide a consistent network of evidence that could be hardly disputed since obtained also in the other various occlusive-studies [6,7,8,9,10]. Thus, at the general and specific level, there is the accuracy of the all of the methods used, and the accuracy of the obtained therapy results. The other limiting argument is the relative paucity of the BPC 157 clinical data [3,4,5]. However, BPC 157 was proved to be efficacious in ulcerative colitis [3,4,5]. This was both in clinical settings [69,70] and in the experimental rats, ischemic/reperfusion vascular ulcerative colitis studies [13] and other ulcerative colitis models [54,55,56,57,58,59], and complications (for review see, e.g., [71]), as in the gastrointestinal lesions in the rats with the occluded superior mesenteric vein as well as in the rats with other vessel, central or peripheral, occlusion [6,7,8,9,10]. A particular point is revealing and applying this concept in practice [72], also in the various species [73], since it has a very safe profile (LD1 could be not achieved) [72], a point recently confirmed in a large study of the Xu and collaborators [74]. There are consistently effective used ranges of BPC 157 (µg-ng) application and used regimens, which may support each other’s effects [3,4,5,71,72], and interestingly, also in the rats with the central venous occlusion, the same beneficial effect of the application, at the swollen brain, intraperitoneally or intragastrically [10] as in the rats with the superior mesenteric vein occlusion. Together, these findings (for review see, e.g., [3,4,5,71,72]) may suggest its physiological role (in situ hybridization and immunostaining BPC 157 in human gastrointestinal mucosa, lung bronchial epithelium, epidermal layer of the skin and kidney glomeruli) [72]. Additionally, BPC 157 is native and stable in human gastric juice after more than 24 h, unlike rapidly degraded standard peptides [72]. In this context, the role of the animal model is indispensable, the practical indicative evidence is even more important. Thus, BPC 157 and major vessel occlusion syndrome were elaborated in this study with particular respect to the superior mesenteric vein injury and therapy of BPC 157. Although we would need additional studies, these rat studies could claim the irremovable occlusion of the superior mesenteric vein as a part of the vessel occlusion-induced perilous syndromes, and the BPC 157 therapy to overwhelm the consequences thereof [6,7,8,9,10]. Likely, this would be relevant for prothrombotic states, surgery, inflammatory bowel disease and malignancy, which are common risk factors for the development of mesenterial vein thrombosis [1].

## Figures and Tables

**Table 1:** The neuropathologic scores.

Brain Area	Grading	Percent Area Affected	Morphological Changes
Cerebral and cerebellar cortex, hypothalamus, thalamus, hippocampus	1	≤10	Small, patchy, complete or incomplete infarcts
	2	20–30	Partly confluent complete or incomplete infarcts
	3	40–60	Large confluent complete infarcts
	4	>75	In cortex; total disintegration of the tissue, in hypothalamus, thalamus, hippocampus; large complete infarcts
Cerebral and cerebellar cortex, hypothalamus, thalamus, hippocampus	1	≤20	A few karyopyknotic of neuronal cells
	2	50	Patchy areas of karyopyknotic areas
	3	75	More extensive of karyopyknotic areas
	4	100	Complete infarction

## Data Availability

The data presented in this study are available on request from the corresponding author.

## Supplementary Materials

The following are available online at https://www.mdpi.com/article/10.3390/biomedicines9081029/s1, Figure S1, supplement.
