# Supplementary material for: Complex Syndrome of the Complete Occlusion of the End of the Superior Mesenteric Vein, Opposed with the Stable Gastric Pentadecapeptide BPC 157 in Rats
Source: Biomedicines. 2021 Aug 17;9(8):1029. doi: 10.3390/biomedicines9081029 (PMC8394093; doi:10.3390/biomedicines9081029)
Supplement: Supplementary file 1 [file biomedicines-09-01029-s001.zip › biomedicines-1154328-supplementary.pdf]

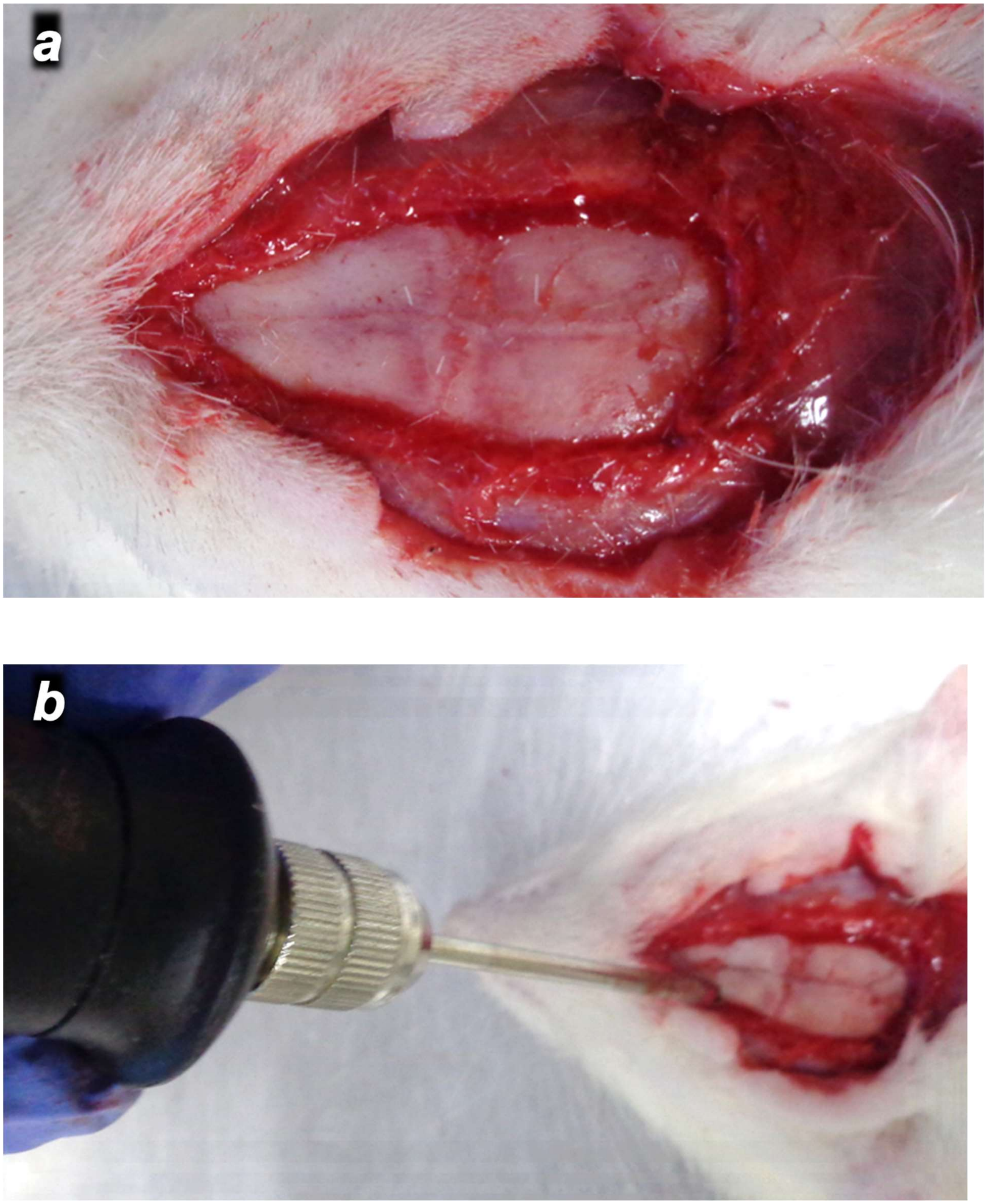
a
b

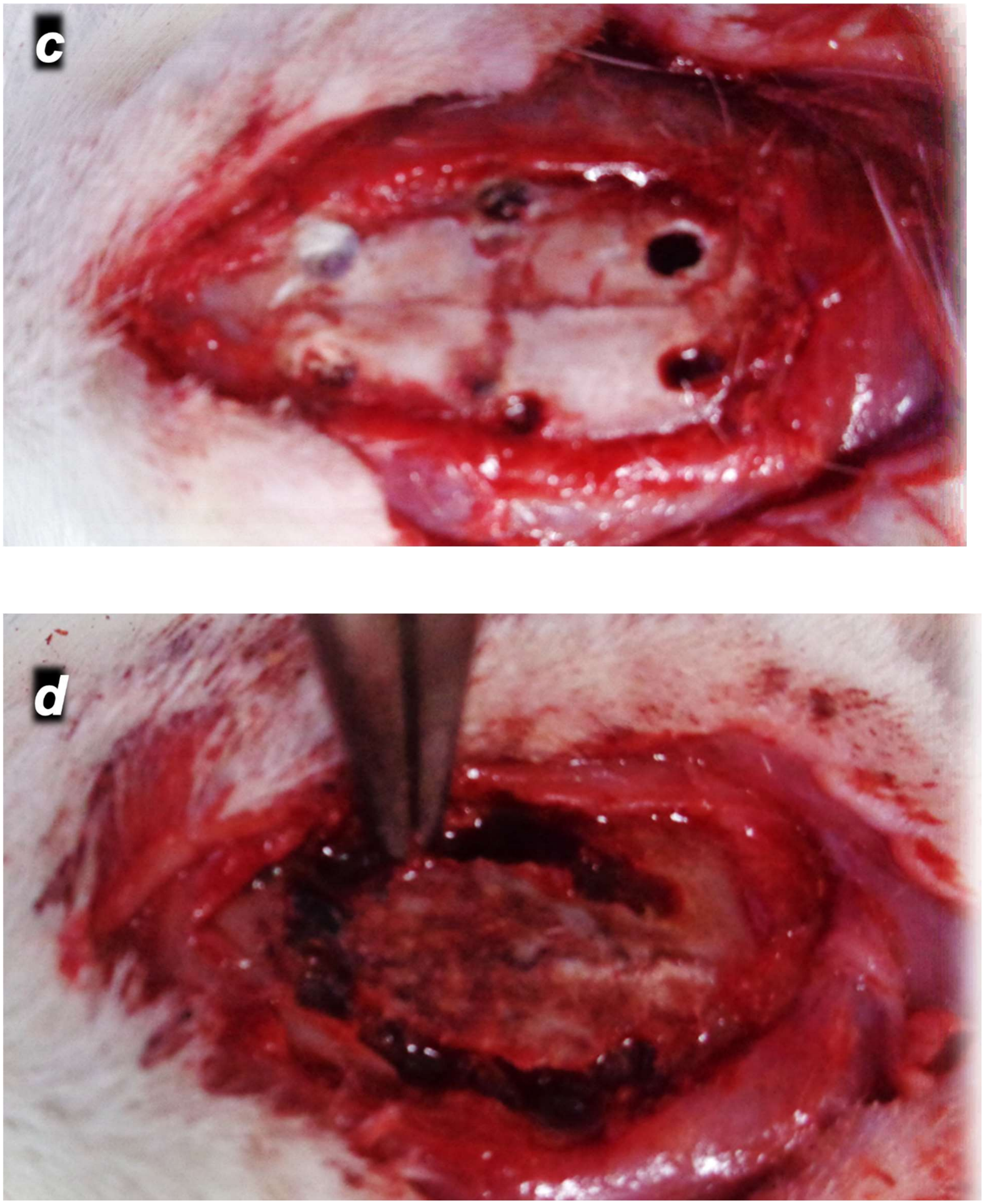
c
d

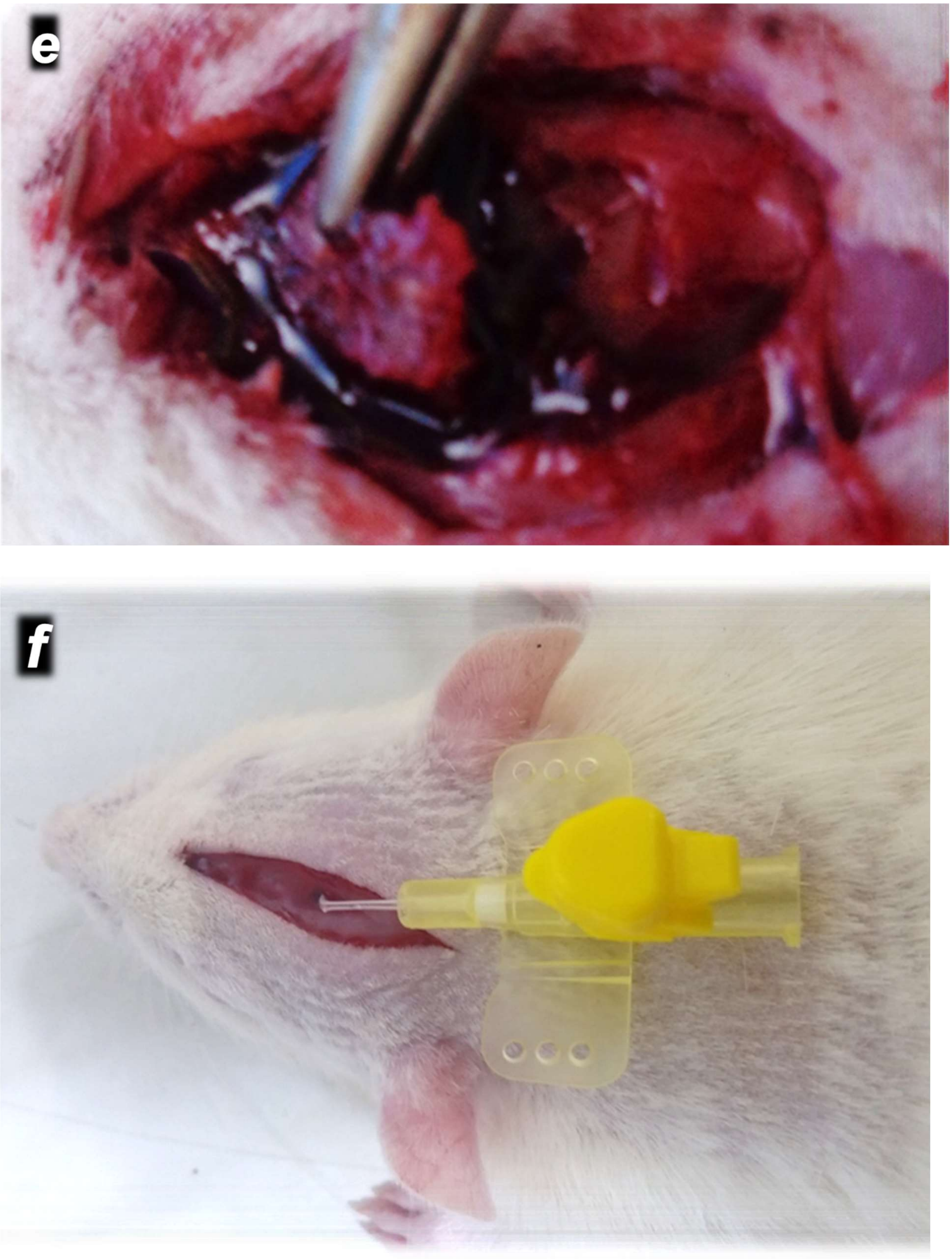
e
f

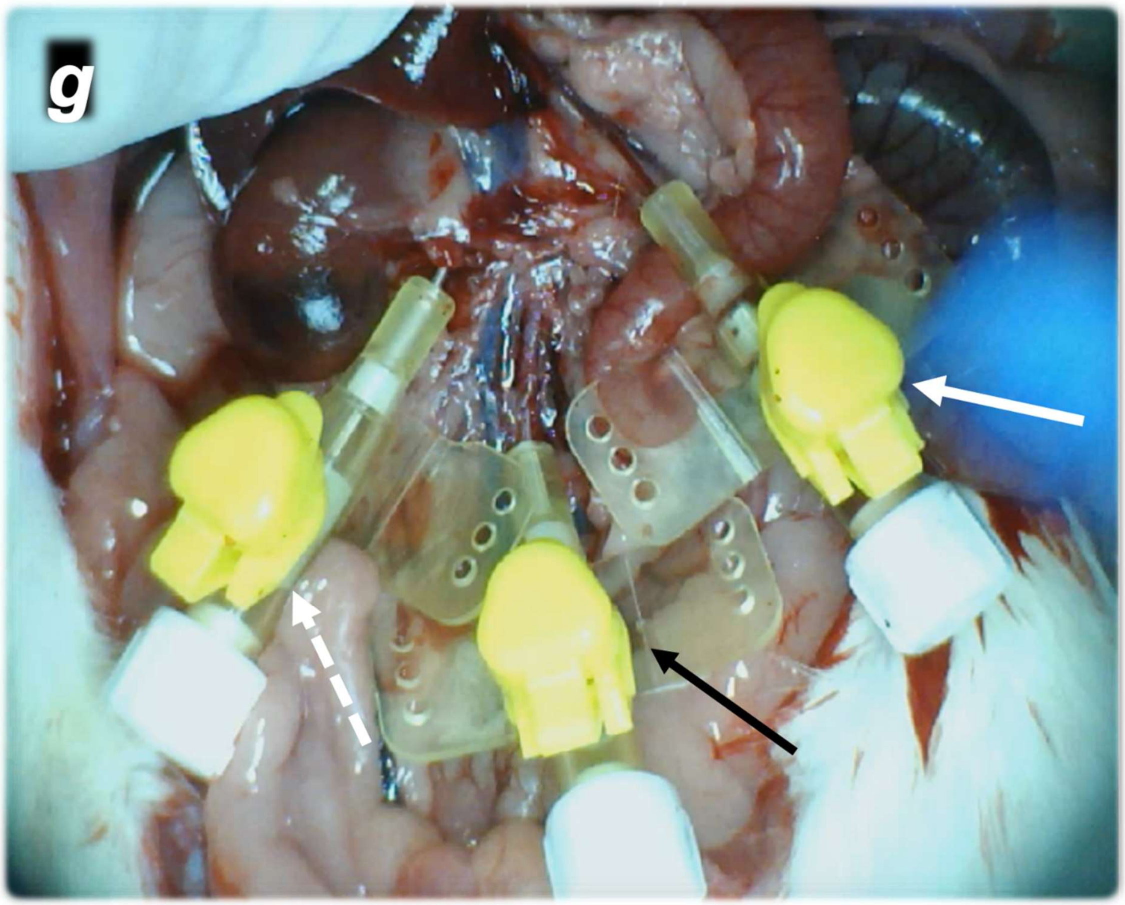
g

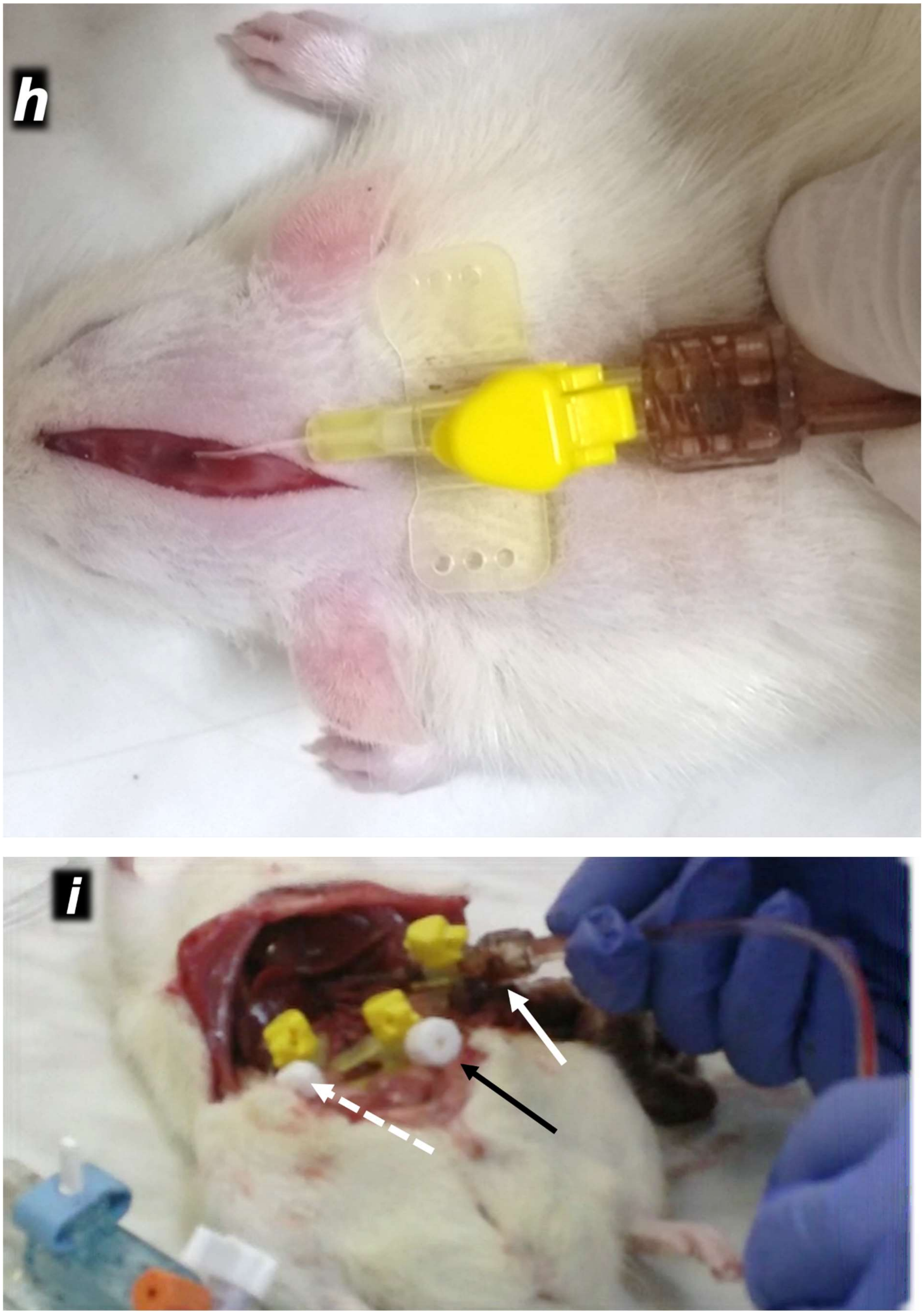

Figure 1. ***a–i***, supplement. ***a–e***. Calvariectomy time sequence to prepare calvarial window for the brain swelling assessment. ***f–i***. As described before [6-10], and presented in the rats with the occluded superior mesenteric vein, recordings were made in deeply anesthetized after with a cannula (BD

Neoflon™ Cannula) connected to a pressure transducer (78534C MONITOR/ TERMINAL; Hewlett Packard, USA) inserted into the superior sagittal sinus, portal vein, superior mesenteric vein and inferior vena cava, and abdominal aorta. Routine presentation of the canulas inserted into superior sagittal sinus (***f***), portal vein (full withe arrow) and inferior vena cava (dashed white arrow), and abdominal aorta (black arrow) in rat (***g***). Superior sagittal sinus canula (***h***) or portal canula connected to a pressure transducer (full white arrow), caval (dashed white arrow) and aortal (black arrow) canulas for further pressure measuring (***i***).
